# Preclinical Potential of Probiotic-Loaded Novel Gelatin–Oil Vaginal Suppositories: Efficacy, Stability, and Safety Studies

**DOI:** 10.3390/gels9030244

**Published:** 2023-03-19

**Authors:** Anchal Bassi, Garima Sharma, Parneet Kaur Deol, Ratna Sudha Madempudi, Indu Pal Kaur

**Affiliations:** 1 University Institute of Pharmaceutical Sciences, Panjab University, Chandigarh 160014, India; 2 G.H.G. Khalsa College of Pharmacy, Gurusar Sadhar, Ludhiana 141104, India; 3 Unique Biotech Limited, Hyderabad 500078, India

**Keywords:** self-preserved formulation, vulvovaginal candidiasis, *Bacillus coagulans*, water activity, viability

## Abstract

The current study describes a suppository base composed of aqueous gelatin solution emulsifying oil globules with probiotic cells dispersed within. The favorable mechanical properties of gelatin to provide a solid gelled structure, and the tendency of its proteins to unravel into long strings that interlace when cooled, lead to a three-dimensional structure that can trap a lot of liquid, which was exploited herein to result in a promising suppository form. The latter maintained incorporated probiotic spores of *Bacillus coagulans* Unique IS-2 in a viable but non-germinating form, preventing spoilage during storage and imparting protection against the growth of any other contaminating organism (self-preserved formulation). The gelatin–oil–probiotic suppository showed uniformity in weight and probiotic content (23 ± 2.481 × 10^8^ cfu) with favorable swelling (double) followed by erosion and complete dissolution within 6 h of administration, leading to the release of probiotic (within 45 min) from the matrix into simulated vaginal fluid. Microscopic images indicated presence of probiotics and oil globules enmeshed in the gelatin network. High viability (24.3 ± 0.46 × 10^8^), germination upon application and a self-preserving nature were attributed to the optimum water activity (0.593 a_w_) of the developed composition. The retention of suppositories, germination of probiotics and their in vivo efficacy and safety in vulvovaginal candidiasis murine model are also reported.

## 1. Introduction

Gelatin is an affordable and widely accessible polymer due to its biodegradability, biocompatibility, and safety in terms of non-toxic byproducts produced upon enzymatic degradation. Gelatin contains motifs such as Arg-Gly-Asp (RGD), which exhibit cell adhesion, ensuring close contact and hence good biological activity as compared to other polymers [1]. This adaptability of the gelatin structure has aroused interest to generate matrices viz. hydrogels, sponges, microparticles, microspheres, sheets, emulsion gels, and nano-carriers [2]. With its good mechanical properties and capacity to provide a solid gelled texture, presently we propose to use it to develop vaginal suppositories. Gelatin is a protein that forms an interconnected, web-like matrix when dissolved in water and creates a structural network in water due to its (i) gelling and (ii) co-emulsifying properties. Not only does the gelatin give the water structure, but it can also suspend other dissolved and undissolved solids in its matrix viz. the probiotic cells. Herein, gelatin coupled with a suitable surfactant in water (aqueous phase) was used to emulsify an oil phase, into which probiotic cells were dispersed. The coupling of gelatin with water prior to the addition of the probiotic dispersed in oil helps to reduce the amount of reactive water. The latter, combined with the coating of the probiotic with oil prior to incorporation into the gelatin solution, doubly protects against germination of the probiotic during storage; the oil coat also maintains probiotic viability. Vulvovaginal candidiasis (VVC) involves inflammation of the vaginal and vulvar epithelium mucosa caused by underlying fungal infection, most commonly with *Candida albicans* and *C. glabrata* [3]. Opportunistic Candida species invade the mucous membrane of the vagina, leading to exuberant mucosal inflammatory responses [3]. Almost 50% of adult women report having experienced at least one episode of VVC when young [4].About 1.4 million outpatient visits per year are reported for vaginal candidiasis, only in the US [5], while the actual number of cases may be much higher, and is not known. Significant drug resistance and VVC recurrence (RVVC) is reported, with conventional antifungal treatments [6] available as vaginal creams, ointments, ovules, and suppositories. In the case of recurrence, physicians may prescribe one to three antifungal drugs to be taken by mouth from two weeks to six months. Fluconazole is the most common drug of choice for oral treatment. Its use is, however, avoided or closely monitored in cases of women taking statins, having kidney disease, or being at high risk for arrhythmias. It is also reported to significantly increase the risk of miscarriage in pregnant women. Topical treatment with a cream or suppository has an advantage of faster onset and freedom from systemic toxicity; however, local irritation is reported with some topical products. Data suggest that recurrent vulvovaginal candidiasis occurs in 6–10% of women [7].There is an expert consensus on topical maintenance therapy with an azole antifungal, or boric acid, at one to three times weekly, following a full-course therapy [8].

Probiotics inhibit pathogenic Candida species and maintain and modulate microbiota profiles in the vagina [6,9,10,11]. Further, they also lower vaginal pH and downregulate inflammatory mediators such as nuclear factor-kappa B (NF-κB), and proinflammatory chemokines and cytokines such as interleukins (ILs) and tumor necrosis factor-alpha (TNF-α)) [11,12,13,14,15]. Probiotics specifically inhibit mitogen-activated protein kinase (MAPK) and pattern recognition receptor (PRR) pathways [16]. The pathways are responsible for the transcription of NF-κB and a range of proinflammatory cytokines, chemokines and other inflammatory mediators [17]. Probiotics are also reported to inhibit Ikb-βα and NF-κβ inhibitor degradation, which ultimately reduces the binding of NF-κB to DNA [18,19].

Delivery of the probiotic through gut to alter vaginal health has previously been reported by many research groups [20,21]. However, the direct delivery of good microbiota to vaginal cells can restore/transplant the host’s indigenous microflora with healthy bacteria and prevent repeat pathogen onslaught. Further, low enzymatic activity in the vagina will ensure that the administered probiotics do not experience harsh conditions in contrast to those observed in the stomach, which can compromise their viability [22]. The manipulation of vaginal microflora by direct administration has thus surfaced recently in a few studies [23,24]. An important consideration with respect to effective probiotic delivery, however, is that the carrier system must maintain the viability and stability of the loaded probiotic, both upon administration and during manufacture and storage. Thus, there are only limited studies on products such as suppositories incorporating whole-cell probiotics for local vaginal delivery. This, according to us, is attributed to manufacturing and storage constraints, a lack of probiotic cell germination in vaginal mucosa post-application, leakiness and limited stay, and the expulsion of products from the vaginal cavity.

Gelatin and glycerol–gelatin vaginal suppositories are extremely popular, attributed to their ease of use and soothing effect, and provide prolonged and complete release of the active load owing to their slow dispersion in mucosal secretions [25,26]. Further, the process involved in the manufacturing of suppositories is simple and scalable without significantly compromising probiotic viability [27]. Stability during storage can be maintained by keeping water activity of the developed system low. When dissolved in hot water, proteins of the gelatin unravel into long strings that interlace when cooled, to create a three-dimensional structure that can trap a lot of liquid. Thus, the developed gelatin–oil-based carrier system was prepared to have low water activity to avoid probiotic multiplication and formulation spoilage during storage [28].

The preservation of the developed system against contaminating organism is another concern with gelatin-based products, as the latter can support the growth of some organisms. Low water activity does not support the growth of such organisms, and thus there is no need to add a preservative into the suppositories. This is of significance for a probiotic product, because the incorporated preservative can affect probiotic viability [28,29].

In the present study, we thus endeavored to develop a suppository system with an intelligent choice of ingredients containing a self-emulsifying gel base, oil, surfactant, and a soothing agent, which keeps the incorporated probiotic viable, prevents any contamination of the product during storage, aids in the germination of the incorporated probiotic upon application, is acceptable for use and helps maintain/transplant healthy flora in the vaginal cavity for vaginal health. *Bacillus coagulans* Unique IS-2, a nonpathogenic, Gram-positive, spore-forming bacteria, was loaded into the above-described oil–gel suppository base. The selected strain of bacteria has reported clinical efficacy against bacterial vaginitis [30]. The developed suppositories were characterized for various pharmacopeial and non-pharmacopeial tests. The suitability of the suppository composition for probiotic delivery was ensured by evaluating its water activity, ex vivo bioadhesion and retention in porcine vaginal mucosa. The anti-candida potential of the probiotic-loaded suppository in terms of inhibition and filament disruption was recorded in vitro. The in vivo performance of the developed suppositories was evaluated in terms of probiotic germination, bioadhesion and retention in the rat vaginal cavity, and the anti-infective effect in comparison to a marketed product (Candid V^®^ gel) in VVC rat model. The developed probiotic–oil–gelatin suppositories were evaluated for stability (2–8 °C for 3 month) and in vivo 15-day safety studies.

## 2. Results and Discussion

As per the official guidelines, there should be uniformity in weight as well as content of the suppositories to elicit an invariable therapeutic effect. The average weight of the developed suppositories was 1.39 g (Supplementary Figure S1). Since only two suppositories differed from the average weight by more than 5% and no suppository differed from the average weight by more than 10%, the formulated suppositories complied with the standards described in the Indian Pharmacopeia (2014) for suppositories. The total probiotic content loaded into the suppositories was found to be 23 ± 2.48 × 10^8^ cfu per suppository. The content uniformity evaluation of the developed gelatin–oil suppositories indicated that the distribution of colony forming units of *Bacillus coagulans* was uniform in the apex and bottom part of the suppository; however, it was considerably low in the middle part (Table 1), indicating that the probiotic needs to be mixed properly after incorporation into the base. The cell count of the used *Bacillus coagulans* powder was confirmed by the pour plate method (*n* = 8) as per the procedure given in the Supplementary Materials. The mean viable count was found to be 21.54 ± 4.51 × 10^6^ cfu/mg. The cell surface hydrophobicity of *Bacillus coagulans* was 30.4 ± 5.34% (Supplementary Table S1); % Hydrophobicity index (% HPBI) is an indicator of adhesive capabilities and a value < 50% is considered low. The results indicated that *Bacillus coagulans* spores were invariably less hydrophobic than their germinated counterparts. The incorporation into the gelatin–oil base thus imparted it adhesiveness until the time the spores germinated.

**Figure 1:**
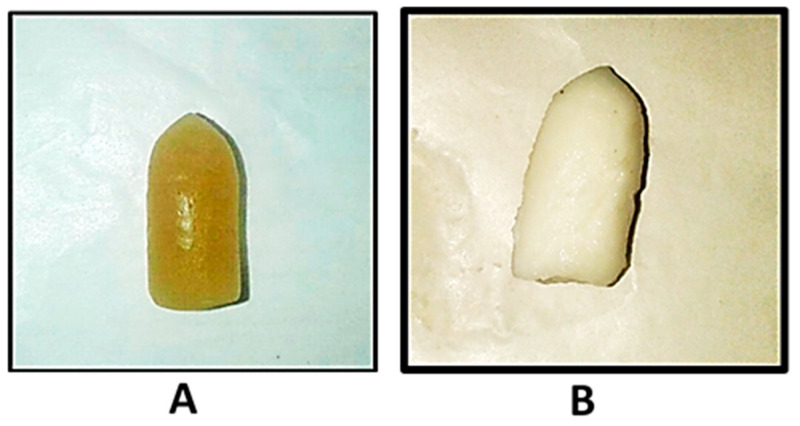
Suppositories after (**A**) 0 h and (**B**) 6 h of swelling (*n* = 3). SVF: simulated vaginal fluid.

The hardness was found to be 1.43 ± 0.175 kg. Gelatin–oil suppositories, due to their elastic nature, are not too firm. It was observed that the suppositories started melting after 10 min (Supplementary Figure S2a) of being placed in the test beaker at 37 °C, and completely melted within 2 h (Supplementary Figure S2b). It was confirmed that the prepared suppositories had a significant capacity to swell, almost to double their initial weight (at 6 h) (Table 2; Figure 1). The swelling behavior of the suppositories was studied at room temperature (25 °C) and the results indicated erosion and dissolution at times after 6 h. However, upon maintaining at 37 °C, fast dissolution within 2 h was recorded. The suppositories completely disintegrated within 2 min. This complies with the Indian Pharmacopeial limits, according to which the water-soluble suppositories should dissolve within 30 min.

**Figure 2:**
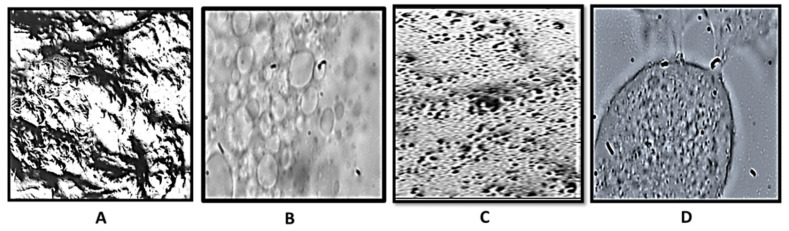
Optical microscopic images of blank ((**A**): 10× and (**B**): 100×) and probiotic-loaded formulation ((**C**): 10× and (**D**): 100×).

Optical images taken at 10 and 100× for blank and probiotic-loaded suppositories (Figure 2) showed a woven/honeycomb network with air voids. Figure 2C shows the presence of probiotic spores enmeshed as small brown clusters. Images taken at 100× depict the gel network more clearly with Figure 2D distinctly illustrating the presence of spores within an oil globule.

**Figure 3:**
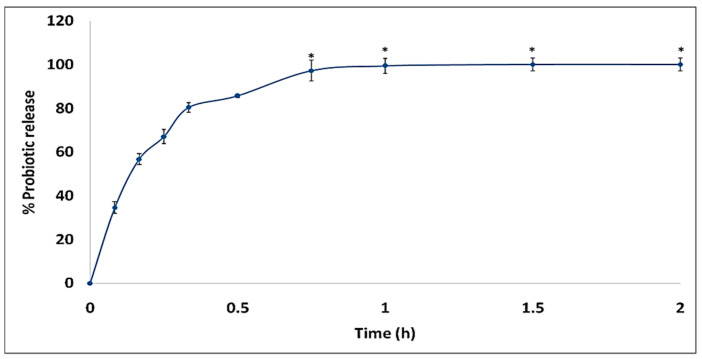
In vitro release profile of probiotic from gelatin–oil suppositories. All values differ significantly except those marked similarly.

Probiotic-loaded suppositories when incubated in 2 mL SVF at 37 ± 0.5 °C resulted in the complete release of probiotics within 45 min, which is well in the limits defined by I.P., 2014 (not more than 60 min) (Figure 3).

**Figure 4:**
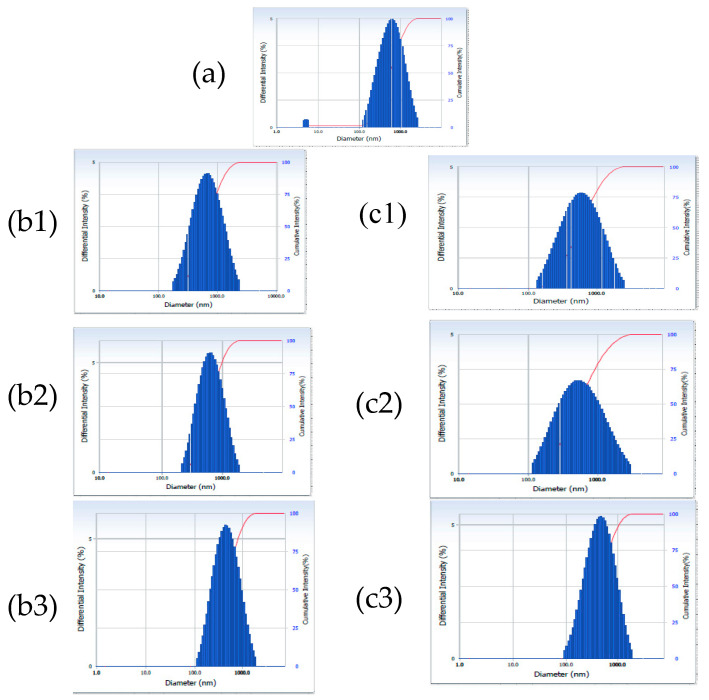
Particle size distribution of blank emulgel suppository dispersed in water (100 mL) at (**a**) zero time, (**b**) after storing at 25 °C and (**c**) 37 °C for 2 h (**b1**,**c1**), 6 h (**b2**,**c2**) and 24 h (**b3**,**c3**), respectively.

**Figure 5:**
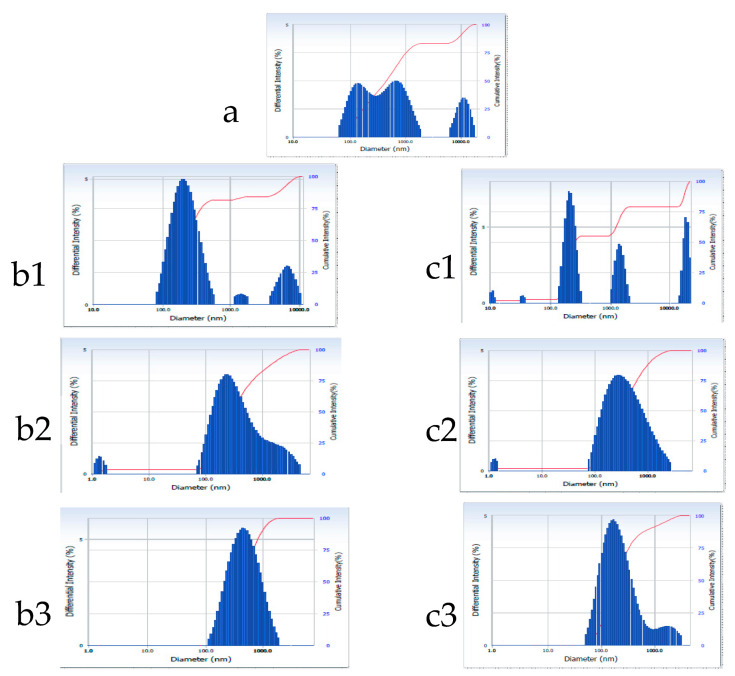
Particle size distribution of probiotic-loaded emulgel suppository dispersed in water (100 mL) at (**a**) zero time, (**b**) after storing at 25 °C and (**c**) 37 °C for 2 h (**b1**, **c1**), 6 h (**b2**,**c2**) and 24 h (**b3**,**c3**).

The size and size distribution of both the blank and gelatin–oil–probiotic suppositories were determined after dispersion in water. At ambient temperature, the average particle size of the blank suppository was found to be 493.1 nm. The distribution curve for the same (Figure 4a) showed a maximum intensity at 800.0 nm and a very small peak observable at <10 nm, which probably shifted the average size to 493.1 nm. The probiotic-loaded suppository recorded an average particle size of 754 nm (Table 3) and a trimodal distribution (Figure 5a). The first peak in case of the probiotic-loaded suppository appeared at 160 nm and may be due to fine oil globules emulsified in the aqueous gelatin base; peak 2 at 753.2 nm may be attributed to gelatin particles; while peak 3 observed at >11,000 nm could be due to the free probiotic or its aggregates in oil globules. Gelatin is known to undergo dissolution upon extended hydration; therefore, studies were continued for 24 h post-dispersion of the suppositories in water at 25 °C (room temperature) and 37 °C (body temperature). The average particle size of the blank formulation remained stable, at both temperatures, for up to 6 h, with a slight reduction at 24 h, attributed to solubility or the erosion of gelatin particles with time. The size distribution curves for probiotic suppository suggested that with passing time the distribution pattern moved from trimodal to unimodal (Figure 5), thus matching that of the blank suppository (Table 3; Figure 4). This could be due to disaggregation of the probiotic with time, attributed to its hydration.

The gelatin–oil suppositories prepared here are expected to release probiotic both by melting and dissolution. Small probiotic-loaded oil particles obtained upon the dissolution of the prepared suppositories will not only cover a larger area but will also achieve an intimate contact with the vaginal mucosa [1]. This will help in the establishment of the probiotic on the mucosal wall.

Water activity (a_w_) is defined as a measure of the energy status of the water present in a sample referred to as free, unbound, or active water. It is different from total water content, as a portion of the total water contained in a sample is at times strongly bound to specific sites of various components in a sample, and hence may not affect the preparation. Active water, on the other hand, influences the shelf life of products as it makes the preparation liable to microbial growth [17,18]. While temperature, pH, and several other factors may also influence the growth of an organism in a product, the rate at which they grow is often monitored by water activity. The lowest a_w_ at which most bacteria will grow in a product is about 0.90. The a_w_ for mold and yeast growth is approximately 0.61 a_w_ with the lower limit for the growth of mycotoxigenic mold being 0.78 a_w_ [19]. The water activity of the freshly prepared suppository was 0.531 a_w_, whereas during storage for two months (2–8 °C) it increased to 0.593 a_w_. Although a marginal increase in water activity was recorded for the developed suppositories, the value was still lower than the reported range (0.61–0.90), favoring microbial (both bacterial and fungal) growth both of the probiotic, or the contaminating organism. Thus, it may be said that the developed gelatin–oil suppository will be non-reactive, or in other words, will keep the probiotic dormant, i.e., without germination (as confirmed in stability studies). Furthermore, suppositories per se will be endowed with a self-preservation against any harmful bacterial growth during storage and use. It may be noted that the suppository base used here was not a simple gelatin or a glycerol–gelatin base; instead, it was a gelatin base incorporating probiotic-laden small oil globules.

**Figure 6:**
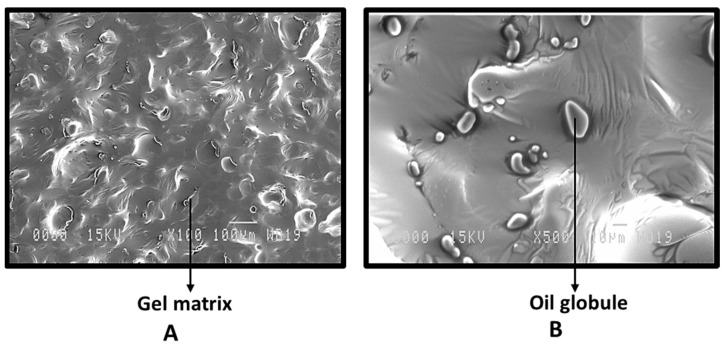
Scanning electron microscopy of *Bacillus coagulans*-loaded suppository (**A**) 100× (**B**) 500× magnification.

SEM pictures of the cross-sectioned suppository confirmed the gel matrix (Figure 6A) interspersed with oil globules (Figure 6B).

The probiotic spores incorporated into the developed formulation were found to maintain their viability (*p* < 0.05) after storage under refrigerated conditions for three months, as shown in Table 4. Obtaining similar values for samples with and without heat shock indicated that the spores did not undergo any germination in the formulation. This is important, as otherwise the cell population in the formulation will keep changing and the growth of vegetative cells may produce materials that foul the suppository, making it aesthetically unpleasant or unfavorable for use.

**Figure 7:**
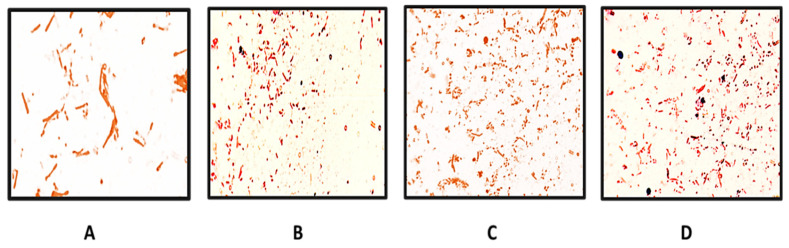
Germination of *Bacillus coagulans* at 100× magnification after (**A**) 2 h, (**B**) 4 h, (**C**) 6 h, and (**D**) 24 h.

*Bacillus coagulans* usually exists as spores, and since the latter are resistant and stable, it was considered appropriate to incorporate and maintain them as spores in the developed formulation. However, to elicit the protective physiological effect, it is important that they grow actively upon insertion into the vaginal cavity. The in vivo germination of the probiotic following the application of the spore-loaded suppository upon insertion into the vagina was established by the swabs collected from the rat’s vagina. The presence of vegetative cells at different time intervals, i.e., 2 h, 4 h, 6 h, and 24 h, were observable on the slides and are suitably labeled in the Figure 7. Spore germination was evidenced from 4 h onwards (Figure 7C) in the samples and abundance was observed, with most of the spores showing germination in the 24 h sample (Figure 7D).

**Figure 8:**
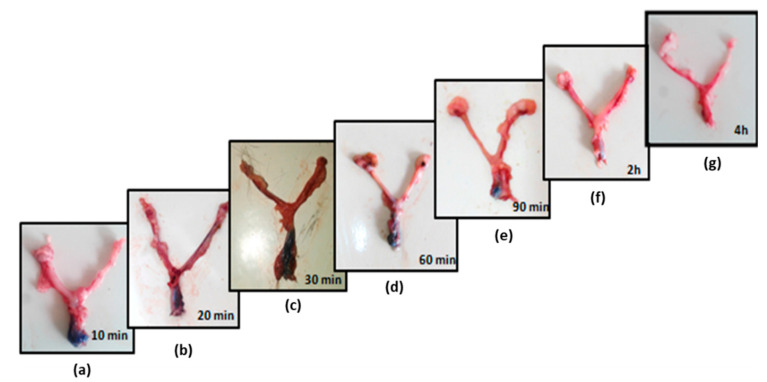
In vivo retention of trypan-blue-labeled suppositories observed at different time points (from (**a**) at 10 min to (**g**) at 4 h) in the dissected vaginal cavity of rats. High-intensity blue color marks the presence of suppository and fading blue color (from 60 min onwards) marks the dissolution/melting of the suppository at the vaginal site.

In vivo retention studies indicated that the suppository was intact and maintained its shape even at 10 min after its insertion into the vaginal cavity of the rats (Figure 8). Complete melting of the vaginal suppository was recorded at the end of 30 min; however, no leakiness was observed through the vaginal opening of the rat (Table 5), indicating a successful formulation. The reasons for this are:

(1) Swelling of gelatin suppositories helps them to snugly fit in the reproductive tract.
(2) Small amount of vaginal fluid is insufficient to initiate the dissolution process, such that the base melts at body temperature to a viscous gel-like consistency, as also observed in the in vitro study. Since the consistency of the melted suppository is high, it stays within the tract and does not leak out.
(3) Furthermore, the release of probiotic-loaded oil particles coated with viscous gel cover a larger vaginal area, with vaginal contractions further helping in a widespread distribution.

The vaginal mucosa is highly sensitive to the exposed chemicals and therapeutic agents that may result in irritation and/or inflammation and can make women more susceptible to various infections. Hence, the vaginal irritation potential of feminine care formulations and vaginally administered therapeutic agents is a significant public health concern. The pH of the developed suppository was found to be 4.3 ± 1.0. The pH is very near the normal vaginal pH, thus the suppositories are expected to be non-irritating to the vaginal mucosa. An in vivo vaginal irritation study was performed in rats to evaluate the safety of the vaginal formulations on their multilayered vaginal epithelium. The latter is a characteristic feature of mammalian vagina, too.

**Figure 9:**
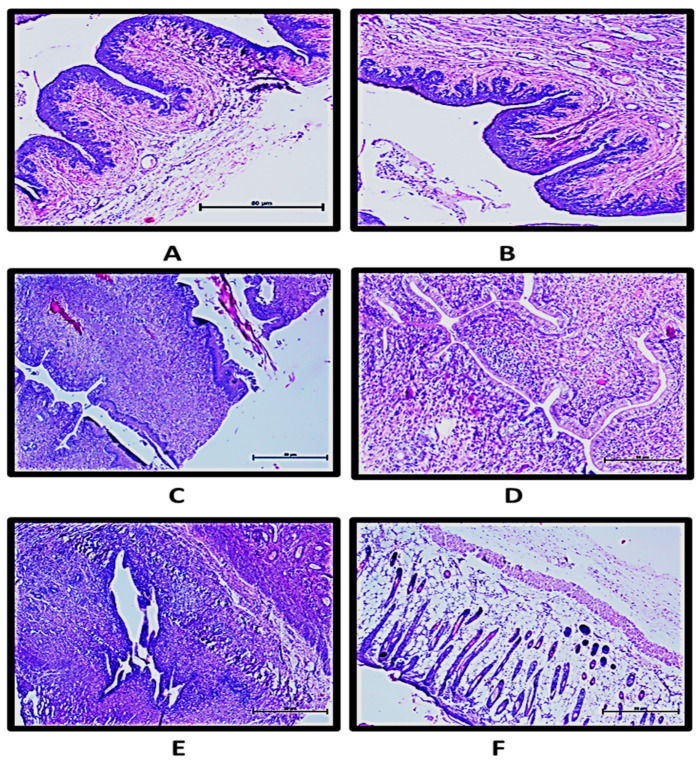
Histology of female reproductive tract of rat showing sections of stratified epithelial layers of (**A**) control and (**B**) treatment groups; cervix area of (**C**) control group and (**D**) treated group; (**E**) uterus of treated group; and (**F**) epithelial layer of treated group.

The results from the histopathological examination (Figure 9) showed that the formulation was well-tolerated by the rat vagina and there were no signs of erythema or edema after 14 days of vaginal administration of the prepared suppositories. It was evident from the images that the epithelial membrane was intact, and no signs of vascular congestion/leucocyte infiltration or edema were observed in the treated groups. Further, the lower cervix, uterus and epithelial layers showed normal histology.

**Figure 10:**
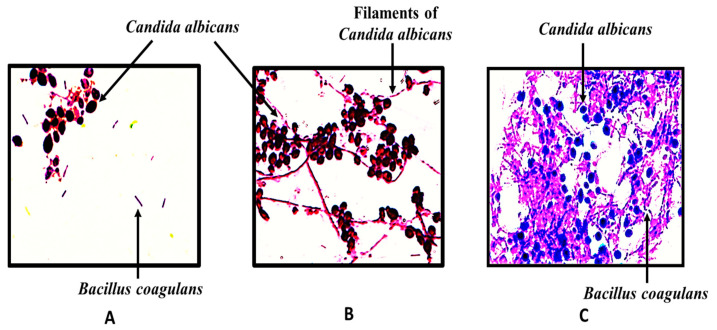
Co-aggregation of *Candida albicans* with (**A**) free *Bacillus coagulans*, (**B**) blank emulgel suppositories (absence of co-aggregates), and (**C**) *Bacillus coagulans* incubated with probiotic-loaded gelatin–oil suppositories.

*Bacillus coagulans* spores are reported to form co-aggregates with *Candida albicans*, and the same was also observed in the present study (Figure 10A). The probiotic spores co-aggregated with the filaments of *Candida albicans*. Visibly better co-aggregation of probiotics from suppository was observed versus free form. Only candida aggregates were observed after incubation with the blank suppository.

**Figure 11:**
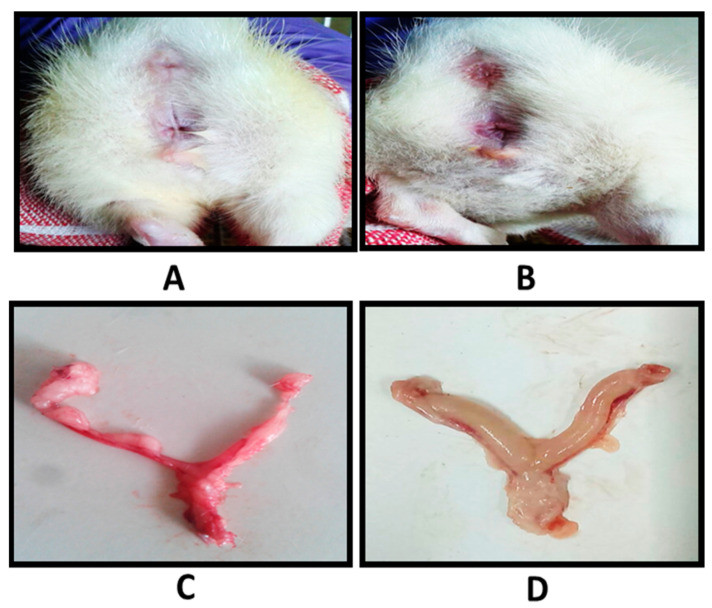
Macroscopic observations of vaginal openings of (**A**) probiotic-loaded suppository treatment group; (**B**) Candida-infected group; the isolated reproductive tracts of (**C**) probiotic-loaded suppository treatment group at day 7, showing healthy pink tissue, and (**D**) Candida infected group with no treatment, showing inflamed tissue.

The in vivo efficacy of the developed formulation was evaluated in a VVC rat model. The establishment of a pseudo-estrous state was confirmed by observing epithelial cells in the vaginal lavage under an optical microscope (Supplementary Figure S3). It was observed that well-formed, nucleated epithelial cells of the pro-estrous stage was replaced by cornified, squamous, densely packed epithelial cells after hormonal treatment. The latter signified the establishment of an estrous phase, after which standardized yeast suspension was introduced into the vagina. Once the Candida infection was confirmed in the animals, scheduled treatments (marketed formulation (Candid V^®^ gel), free probiotic suspension, probiotic-loaded suppositories, and blank suppositories) were initiated in the infected rats and the fungal burden was monitored every day for seven days. Macroscopic observation (Figure 11) indicated redness of the vaginal opening and inflammation of the reproductive tract of animals infected with *Candida albicans*. Correspondingly, probiotic-loaded-vaginal-suppository-treated animals showed significant improvement during the experimental period.

The probiotic-loaded suppositories did not show any inhibition by either well diffusion or the streak method. It may be concluded that the vegetative probiotic cells do not inhibit *Candida albicans* in vitro probably because (a) the inhibition follows mechanisms other than the release of bacteriocins/inhibitory substances in the surrounding media; (b) factors such as the immune responses of the host, changes in the immune system induced by *C. albicans* enzymes or metabolites present in the vaginal lumen cannot be mimicked in the in vitro tests, although they do exert a great influence on the effects demonstrated by probiotics in vivo.

**Figure 12:**
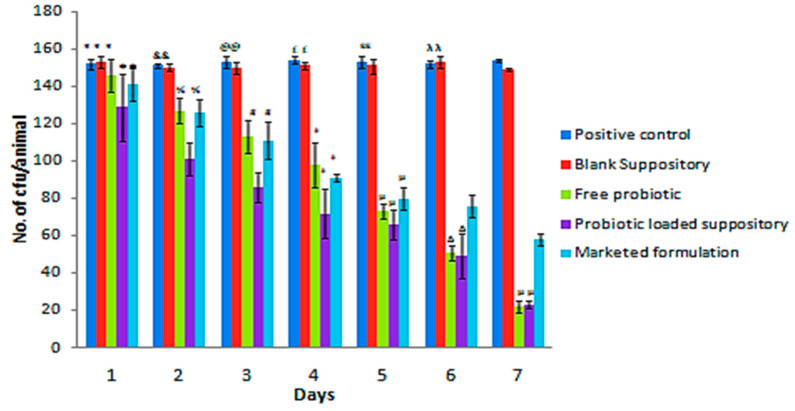
Effect of various treatments every day for seven days on the reduction in cfu of Candida in the rat model of vulvovaginal candidiasis (*n* = 3). All groups differ significantly except those marked similarly (intraday comparison between different groups) at *p* < 0.001.

Fungal burden studies (Figure 12) indicated that the positive control (infected) group was not significantly different from the blank-suppository-treated infected group, indicating that the suppository base per se does not elicit any physiological effect. Further, in the initial phase of treatment, no significant difference (*p* < 0.001) was observed between the marketed formulation and the free-probiotic-treated group, whereas the probiotic-loaded suppository group showed significantly (*p* < 0.001) better results. However, on the 7th day of treatment, both the free probiotic group and probiotic-loaded suppository group showed significantly better effects than the marketed formulation. The free probiotic dispersion showed significant effects; however, it tended to leak out following application, clearly concluding that its incorporation into a suppository base, designed herein, is a successful dosage form.

Furthermore, as is expected of azole antifungals, which are fungistatic in nature, the marketed formulation containing clotrimazole did not show any significant decrease in fungal loads from day four onwards, while the decrease in fungal loads continued with both the probiotic-treated groups. The cfu/animal for the positive control group and blank suppository group did not show any significant inter-day difference, while for the free probiotic group and probiotic suppository group, a significant difference was observed. In the marketed formulation, no significant difference was observed between the 1st and 2nd day and 4th, 5th, and 6th days (Figure 12). What is interesting to note is the fact that although *B. coagulans* spores and vegetative cells did not show any activity against Candida in vitro, the effects obtained in vivo establish the usefulness of both the probiotic per se and the developed gelatin–oil suppository formulation. Thus, it may be said that the observed in vivo effects are mediated by mechanisms such as the competitive exclusion of adherence to epithelial cells by Candida, co-aggregation and auto-aggregation leading to the inhibition of Candida, and the modulation of both the immunological reactions initiated by Candida and the local immunological host response.

The in vivo translocation study indicated that only 0.007 ± 0.001% of 122 × 10^6^ spores, administered per day for seven days, translocated to reach systemic circulation when inserted into the infected animals. This value is clearly insignificant, and it was confirmed that the presently used strain was safe for vaginal administration in infected animals. It confirms that the action of the developed probiotic-loaded vaginal suppositories remains localized.

## 3. Conclusions

In the present study, we developed probiotic-loaded gelatin–oil biphasic suppositories for combating vaginal fungal infections. The formulation was tested in an animal model of vulvovaginal candidiasis where it showed effects comparable to a marketed product (Candid V^®^ gel). Unlike the available vaginal probiotic products in the market that contain either extracellular substances or bioactives obtained from probiotics, the currently presented formulation consists of whole-cell probiotics.

The probiotic-loaded vaginal gelatin suppositories described here addressed the stability and viability issues associated with the incorporation of live probiotic cells into the delivery system, not only during production but also storage. The gelatin, due to its intrinsic nature, can keep reactive water to a minimum to avoid the germination of *Bacillus coagulans* spores to their active vegetative form during storage.

Confirmatory proof of the germination of probiotic spores into vegetative form upon insertion of the suppository into the rat vagina was demonstrated in the study. Not only the viability, but the germination of the probiotic spores on the application site is an important consideration because the purported therapeutic effects such as the release of various enzymes, bacteriocins and metabolites (bioactives) are demonstrated by these strains in their active state.

The developed gelatin–oil suppositories were safe in terms of pH, vaginal irritation, and translocation/permeation. High patient acceptability is expected, as indicated by in the vivo retention study suggesting no leakiness.

The in vivo therapeutic effects of the probiotic-loaded suppositories were confirmed in terms of the decrease in the cfu of Candida in infected rats. The developed probiotic-loaded suppositories can also incorporate other probiotic cells, including their vegetative forms, with equal success, as they have the capability of keeping the loaded probiotic in its dormant state during storage. Furthermore, the components and equipment used in this study are cheap and widely available, which makes it an industrially viable option.

## 4. Materials and Methods

### 4.1. Materials

*Bacillus coagulans* (Unique IS-2; MTCC 5260) (100 × 10^6^ cfu/mg) was provided by Unique Biotech Ltd., Hyderabad, India. Gelatin (Qualigens Fine Chemicals Pvt Ltd., Mumbai, India) was light yellow to beige in color having pH of 5–6 (1% solution), and gel strength of 156 G bloom. Glycerin (LobaChemie Pvt Ltd., Mumbai, India), Tween 80^®^ (CDH (P) Ltd., New Delhi, India) and sunflower oil (Amrit Banaspati Company Ltd., Chandigarh, India) (linoleic acid 40–80% of unsaturated fatty acids) were used for preparation of probiotic formulations in the present study.

All the microorganism cultures employed in this study were procured from Institute of Microbial Technology (IMTECH), Chandigarh, India. Culture medium including PNY, agar, Sabouraud and soyabean casein digest were purchased from Hi Media Laboratories Pvt Ltd., Mumbai, India.

### 4.2. Preparation of Bacillus coagulans-Loaded Vaginal Suppositories

Gelatin was dissolved using a magnetic stirrer in the aqueous phase comprising a suitable surfactant and humectant at 60 °C to result in a gel-like matrix. Weighed quantity of probiotic spores was dispersed in sunflower oil, maintained at 60 °C, which was then emulsified slowly with the aqueous phase. The mixture was cooled to 45 °C and poured into previously cooled suppository molds. Suppository-loaded molds were refrigerated overnight, and the formed suppositories were removed, packed in butter paper, and stored in tightly closed containers until further use.

### 4.3. Evaluation of Developed Probiotic-Loaded Gelatin Suppositories

#### 4.3.1. Uniformity of Weight

Variation in weight of suppositories was recorded by weighing 20 suppositories and calculating average weight.

#### 4.3.2. Hardness

The test is used to measure the mass (in kilograms) that a suppository (*n* = 6) can bear without breaking. It also determines the tensile strength of the suppositories to access whether they will be able to withstand the wear and tear during transportation and storage. Hardness of the suppositories was evaluated using Monsanto hardness tester.

#### 4.3.3. Melting Time of Suppositories

The suppositories (*n* = 6) were kept in closed containers over a water bath at 37 °C and observed for the lag time before the melting started and the time taken to melt completely.

#### 4.3.4. Swelling Studies

Extent of swelling of probiotic-loaded suppositories (*n* = 6) was determined by placing a pre-weighed suppository in a 50 mL beaker at room temperature. Fixed quantity (0.75 mL) of simulated vaginal fluid (SVF; composition described in Supplementary Table S2) was added to each suppository at regular interval of 15 min up to 1 h. After 1 h the suppository was taken out, dried with the help of filter paper to remove fluid present on its surface and reweighed. The same procedure was repeated for 6 h.

#### 4.3.5. Optical Microscopy

The probiotic–oil–gelatin matrix was observed by spreading a small portion of prepared base as a thin smear over a glass slide and observing it at 10× and 100× magnification (Zeiss, Axio A1, Deutschland, Germany).

#### 4.3.6. Disintegration Test

Suppositories were placed in disintegration apparatus containing 1000 mL of SVF and maintained at 37 ± 0.5 °C. The time taken by the suppository to either melt or dissolve completely in the medium was taken as the disintegration time (Indian Pharmacopeia, 2014).

#### 4.3.7. Total Probiotic Content

Suppositories (*n* = 6) were dissolved in 10 mL of sterile saline solution, one at a time, and diluted appropriately (10^5^–10^6^). The dilutions (1 mL) were subjected to heat shock for 30 min (to kill any vegetative cells) at 75 °C and plated on sterilized PNY agar medium [15]. The plates were incubated aerobically at 37 °C for 48 h and the colonies thus formed were counted. Plates containing 30–300 cfu were considered to interpret the results. Total probiotic content was calculated by the given equation:

(1) $$\mathrm{Total}\;\mathrm{probiotic}\;\mathrm{content}\;\left(\%\right)=\;\frac{\mathrm{cfu}\;\mathrm{obtained}\;\mathrm{from}\;\mathrm{the}\;\mathrm{suppository}}{\mathrm{cfu}\;\mathrm{that}\;\mathrm{should}\;\mathrm{be}\;\mathrm{ideally}\;\mathrm{present}}\;\times 100$$

The test was performed in triplicate (*n* = 3).

#### 4.3.8. Content Uniformity

The suppository was cut into three parts: apex, middle, and bottom portion. Each part was weighed and dissolved completely in 10 mL of normal saline solution (0.9% *w*/*v*). Samples were diluted appropriately (10^6^) and underwent heat shock at 75 °C. After cooling to about 45 °C, 1 mL aliquots were plated on previously prepared PNY agar medium. The colonies were counted, and results expressed as cfu/g for each part and compared with one another to confirm uniformity of distribution. The test was performed on 6 suppositories.

#### 4.3.9. Dissolution Study

In vitro dissolution of developed suppositories was carried out in SVF to study probiotic release behavior. The suppositories were kept in closed sterile containers at 37 ± 0.5 °C followed by incremental addition of 2 mL SVF every 15 min for 2 h. At definite times, SVF was withdrawn, diluted appropriately, and 1 mL aliquots were plated on previously prepared sterile PNY agar plates.

#### 4.3.10. Size and Size Distribution

The size and size distribution of both blank and probiotic-loaded suppository dispersion (diluted 100 times) were determined by dispersing each suppository in water. Dynamic light-scattering method (DLS), using a computerized inspection system (Beckman Zetasizer, Delsa Nano C, Switzerland) was used to determine size and size distribution at 25 °C and 37 °C. The light was emitted from a laser diode (658 nm, 30 mW) at an angle of 160°.

#### 4.3.11. Zeta Potential

The measurements for both probiotic-loaded and blank probiotic dispersions were performed at 25 °C using Delsa Nano C, Beckman Coulter at the electric field strength of 23.2 V/cm. The zetasizer measures the zeta potential based on the Smoluchowski equation:

ζ = UE η/ε

where ζ is zeta potential, UE is electrophoretic mobility, η is viscosity of the medium and ε is dielectric constant.

#### 4.3.12. Water Activity

Water activity of freshly prepared suppositories and those stored for 2 months at 2–8 °C for the months May and June 2016 (Chandigarh 30.75000N, 76.78000E ICH Zone IV, India) was determined with a computer-controlled Rotronic Hygro Lab (Rotronic AG, Bassersdorf, Switzerland), operating between 0.5 and 50 °C. The sample was placed in a sealed container that slowly exchanged moisture with the air inside the container until equilibrium was reached. The equilibration process was monitored by measuring the humidity of the air above the sample with a relative humidity sensor.

#### 4.3.13. Scanning Electron Microscopy

A cross-section of the developed vaginal suppositories was examined using a scanning electron microscope (Model JSM6100 (Jeol) with Image Analyser). Prior to observation, samples were dried under a vacuum drier and were mounted on metal grids using double-sided adhesive tape.

### 4.4. Stability Studies

The suppositories were evaluated in terms of change in viability of the loaded probiotic formulation and physical appearance upon storage under refrigerated conditions, i.e., 2–8 °C for 3 months.

### 4.5. In Vivo Studies

#### 4.5.1. Animal Used

Sprague Dawley female rats (150–200 g) obtained from central animal house, Panjab University, Chandigarh, India were used for the study. The animals had free access to standard rodent food pellets (Ashirwad Industries, Mohali, India) and water. They were acclimatized to the laboratory conditions before the experiment. The experimental protocols were approved by the Institutional Animal Ethics Committee (IAEC) (approval no. PU/IAEC/S/15/03) of Panjab University, Chandigarh, India.

#### 4.5.2. Preparation of Mini Suppositories for Use in Rats

The previously described suppository base was poured into small molds prepared using aluminum foil and kept in the refrigerator overnight for solidification. The size of molds was such that it would form suppositories of a size suitable for insertion into the rat vagina.

#### 4.5.3. Germination Study

The vaginas of rats were sterilized by washing with sterile PBS solution followed by gavage with 200 µL of 70% alcohol. The opening of the vagina was also washed with 70% alcohol to kill any adhering bacteria. After 1 h, the rat’s vagina was again washed with sterile PBS solutions. The suppositories were inserted into the rat’s vagina followed by swabbing at 4 h, 6 h and 24 h post-insertion. The swabs were rolled onto the glass slides followed by their microscopy. The heat-fixed slides were treated with malachite green (5%) for 5 min in a hot boiling water bath. After 5 min of heating, excess stain was removed by washing under running water. This was followed by the addition of safranin stain to the slides for 30 s and washing off excess stain under running water and blot drying. Stained slides were examined at 100× in oil-immersion lens by light microscopy (Zeiss, Axio A1, Deutschland, Germany). Germination of the spores was determined by distinguishing the pink color of vegetative rods from green spores.

#### 4.5.4. Retention Studies

The trypan-blue (0.4% mixed in the aqueous phase)-loaded dark-blue-colored suppositories were prepared and inserted into the rat vagina with the help of forceps. The animals (*n* = 3) were sacrificed by cervical dislocation at different time points (10 min, 20 min, 30 min, 60 min, 90min, 120 min and 240 min), followed by dissection to observe vaginal cavity and its scoring in terms of intactness of suppository and intensity of blue color at the site, and leakiness, if any.

### 4.6. Vaginal Irritation Study

This study was planned to evaluate the developed suppositories for cervico-vaginal toxicity and inflammation at the cellular and tissue levels [31]. The experiment was approved by Institutional Animal Ethical Committee (IAEC), approval no. PU/IAEC/S/15/03. Mini suppositories (as discussed previously in Section 4.5.2) with the required dose as per average weight of the animals were prepared for insertion into rat vagina. Animals (Sprague Dawley female rats (150–200 g)) were divided into two groups, i.e., control (untreated) and those administered with probiotic suppositories, with three animals in each group. Probiotic-loaded suppositories were inserted into the vaginal cavity of rats (*n* = 3) for 14 consecutive days while the control group (*n* = 3) was administered with only SVF (20 µL) during these times. The rats were observed for any apparent signs of redness, swelling, vaginal discharge or bleeding every day for 14 days. On the 15th day, animals were euthanized; vaginal tissues were excised and fixed in 10% formalin. Tissues were embedded in paraffin and sections were cut using microtome. The sections were stained with hematoxylin and eosin. Each of the three regions, upper (cervico-vagina), middle, and lower (uro-vagina) vagina, was examined for: (i) epithelial exfoliation, (ii) vascular congestion, (iii) leukocyte infiltration, and (iv) lamina propria thickness/edema. Sections were scored (as explained in Supplementary Table S3) to define vaginal inflammation.

### 4.7. Coaggregation Studies

Free *Bacillus coagulans* as well as that loaded into suppositories was tested for its ability to co-aggregate with the *Candida albicans*. The probiotic suppository was dispersed in 10 mL of normal saline (0.9% *w*/*v*) solution. Next, 1 mL of free probiotic suspension, or that obtained from suppository, (10^8^ cfu mL^−1^) was mixed with 1 mL of *Candida albicans* suspension (10^7^ cfu mL^−1^) on a vortex mixer for at least 10 s and then co-incubated for 24 h at 37 °C and kept under agitation using an incubator shaker. A drop of the suspension was then placed on a glass slide and Gram-stained for visual observation of aggregates. The detailed method is described in the Supplementary Data.

### 4.8. In Vitro Inhibition of Candida Albicans by Probiotic-Loaded Vaginal Suppositories

In vitro inhibition studies of probiotic-loaded vaginal suppositories against *Candida albicans* were performed by two methods:

#### 4.8.1. Well Diffusion Method

The appropriate cell density of *Candida albicans* from cell suspension was spread on already prepared PNY plates to achieve 10^5^ cfu/mL. Wells of 4 mm were formed, a dispersion of probiotic formulation (200 µL) containing 10^8^ cfu/mL was added into the prepared wells, and plates were incubated at 37 °C for 48 h.

#### 4.8.2. Streaking Method

The appropriate cell density of Candida albicans cells from cell suspension was streaked at the center of the already prepared PNY plates at a cell density of 10^5^ cfu/mL. Probiotic dispersion was streaked on both sides around *Candida albicans*, and plates were incubated at 37 °C for 48 h.

### 4.9. In Vivo Evaluation of Bacillus Coagulans-Loaded Suppositories in VVC Rat Model

#### 4.9.1. Animals Used

Sprague Dawley female rats (150–200 g) obtained from central animal house, Panjab University, Chandigarh, India were used for the study. The animals had free access to standard rodent food pellets (Ashirwad Industries, Mohali, India) and water. They were acclimatized to the laboratory conditions before the experiment. The experimental protocols were approved by the Institutional Animal Ethics Committee (IAEC) (approval no. PU/IAEC/S/15/03) of Panjab University, Chandigarh, India.

#### 4.9.2. Establishing the Pseudo-Estrous State

Animals were subcutaneously injected with hormonal injections (β-Estradiol) at a dose of 25 mg kg^−1^ every day for 6 days. On the sixth day lavage was performed using a plastic pipette using 20 µL of sterile saline. The vaginal lavage was smeared on glass slides and observed using optical microscope for the presence of round nucleated epithelial cells, cornified irregular enucleated cells, and small round leukocytes. The proportions of these cells were used to determine the phase of the estrous cycle, and to establish the onset of the pseudo-estrous phase of the estrous cycle of the rats just prior to the first hormonal administration was also determined, through the same procedure.

#### 4.9.3. Standardization of the Concentration of the Yeast Suspension for Infection

Pure culture of *C. albicans* (ATCC 90028) was procured from the Institute of Microbial Technology (IMTECH), Chandigarh, India, and maintained on yeast extract–peptone–dextrose (YPD) agar slants at 4 °C. A single colony from YPD agar plate was inoculated in 50 mL of YPD broth and incubated at 37 °C for 24 h. Cells from the activated culture were aseptically collected by centrifugation at 2000× *g* for 5 min, washed thrice and resuspended in PBS (pH 7.4). The stock cell suspension was serially diluted and cell density from a suitable dilution was measured using a hemocytometer. Cell suspension with a suitable cell density (10^5^ cfu/mL) was used for further experimentation.

#### 4.9.4. Vaginal Infection in Rats

First, 20 µL (10^5^ cfu/mL) of the previously standardized yeast suspension was introduced into the vagina 1 week after hormonal induction. The hormone injections were continued throughout the study period, but on alternate days. The same volume of sterile saline was used for the control rats. A vaginal lavage was taken two days after the challenge and inoculated onto YPD plates supplemented with chloramphenicol (50 mg mL^−1^) to confirm the establishment of infection. The infection was considered positive if at least one yeast colony grew for an individual counting, and a mean count for all the cultures from each rat was ≥10 cfu/mL. The same procedure was followed for the rats from the control group. Thereafter, one suppository was administered intravaginally every day to the treatment groups (Table 6). Vaginal lavages were collected by washing with 100 µL saline 6 h after administration. Samples were appropriately diluted, and spread on YPD plates supplemented with chloramphenicol and incubated at 37 °C for 48 h. Thereafter, cfu/mL was counted and a graph of log cfu/mL versus time was plotted. The study was conducted in comparison with marketed product (Candid V^®^ gel) for a period of 7 days and the decline in fungal count was compared using the *t*-test.

#### 4.9.5. Translocation Study

At the end of 7-day treatment, blood samples were withdrawn from retro-orbital plexus of these animals and collected into sterile microcentrifuge tubes. The collected blood was spread upon the previously prepared PNY agar medium plates with the help of a spreader. Plates were incubated at 37 °C for 48 h and the colonies thus formed were counted. Similarly, the spleen and liver of these animals were isolated aseptically after sacrifice and the tissue homogenates (%*w*/*v*) were plated to confirm (cfu/plate) the translocation of any probiotic to the spleen and liver of the rats. The whole procedure was performed aseptically.

### 4.10. Statistical Analysis

The data was processed statistically using ANOVA (Prism 6.01 GraphPad Software). The *p*-values for each study are indicated for the respective figures of tables.

## Figures and Tables

**Table 1:** Content uniformity within the suppositories.

S. No.	Portion of the Suppository	Probiotic Content Found (cfu/g)
1	Apex	33.81 ± 3.50 × 10^8^
2	Middle portion	24.30 ± 1.35 × 10^8^
3	Bottom portion	39.80 ± 1.90 × 10^8^

**Table 2:** % weight gain of suppositories in SVF.

Time of Swelling (h)	% Weight Gain ± S.D
2	35.86 ± 8.61
3	72.16 ± 23.63
5	96.13 ± 19.39
6	103.87 ± 9.23

**Table 3:** Particle size of probiotic-loaded vaginal suppositories dispersion.

Time (Hours)	Particle Size (nm)			
	At 25 °C		At 37 °C	
	Blank	Probiotic	Blank	Probiotic
0 *	493.1	754.0	-	-
2	555.5	615.1	495.0	994.4
6	558.9	336.1	481.3	314.3
24	397.8	245.0	383.9	374.7

* zero-hour samples were at ambient temperature.

**Table 4:** Cell viability (in cfu) of developed formulation at different time points.

Storage Condition	Zero Day	1 Month	2 Months	3 Months	No Heat Shock 3 Months
0–4 °C	23.1 ± 2.48 × 10^8^	23.6 ± 0.20 × 10^8^	23.4 ± 0.31 × 10^8^	23.9 ± 0.46 × 10^8^	24.3 ± 0.46 × 10^8^

No statistically significant difference was observed (*p* < 0.05) in any value at various times with and without heat shock.

**Table 5:** Scoring of in vivo retention of formulated suppositories.

Time Point	Intactness of Shape	Leakiness
10 min	+++	-
20 min	++	-
30 min	+	-
60 min	-	-
90 min	-	-
2 h	-	-
4 h	-	-

(+++) Suppository maintained its shape; (++) suppository slightly distorted; (+) suppository converted into gel; (-) no leakiness.

**Table 6:** Various groups planned in the study (*n* = 3).

Group No.	Treatment Received
I	Naive control
II	Positive control
III	Blank suppository
IV	Probiotic-loaded suppository
V	Free probiotic suspension in 1% CMC
VI	Marketed preparation (Candid-V gel^®^)

CMC: Carboxymethyl cellulose.

## Data Availability

The submitted data forms a part of master’s thesis and has not been published elsewhere.

## Supplementary Materials

The following supporting information can be downloaded at: https://www.mdpi.com/article/10.3390/gels9030244/s1, Figure S1: Weight of the emulgel suppository versus % difference from average; Figure S2: Melting of emulgel suppositories placed at 37 °C (a) Onset at 10 min (b) Complete melting at 2 h; Figure S3: Vaginal smear showing nucleated epithelial cells in rats prior to estrogen treatment (A) 10×, (B) 100×, and after 6 days of estrogen treatment: (C) 10×, (D) 100×; Table S1: Criteria for categorizing the hydrophobicity of bacteria; Table S2: Composition of simulated vaginal fluid; Table S3: Grading of inflammatory reactions of vagina.
