# Supplementary material for: Preclinical Potential of Probiotic-Loaded Novel Gelatin–Oil Vaginal Suppositories: Efficacy, Stability, and Safety Studies
Source: Gels. 2023 Mar 19;9(3):244. doi: 10.3390/gels9030244 (PMC10048646; doi:10.3390/gels9030244)
Supplement: Supplementary file 1 [file gels-09-00244-s001.zip › gels-2179110-supplementary.pdf]

Material and Methods

1. Establishment of cell count of the lyophilised culture of B*acillus coagulans* used in the study

Exactly weighed quantity (10 mg) of the lyophilised culture of *B. coagulans* obtained from Unique Biotech Ltd., Hyderabad, India, was suspended in 10 ml sterile saline solution, mixed gently and kept at room temperature for 5 minutes to hydrate. The suspension was vortexed for 5–7 minutes, and diluted serially to a decimal dilution of 10^6. The final dilution tubes (10^5–10^6) were allowed to stand in a water bath at 75ºC for 30 minutes (heat shock- for spore selection and to kill any vegetative cells), after which the tubes were cooled to 45ºC. The samples were re-vortexed for 30 sec and an accurately measured quantity (1ml) was transferred to sterile plates. About 25 ml of PNY-agar medium previously sterilised and cooled was added to the plates and the media and culture were mixed as per milk marketing board dilution method (Gunn, 2000). The plates were incubated aerobically at 37 ºC for 48 h and the formed colonies were counted. Plates containing 30–300 cfu were considered to interpret the results.

2. Hydrophobicity of *Bacillus coagulans* culture:

10 mg of lyophilised bacteria was dispersed in 10 ml saline solution and the optical density (OD) of the bacterial suspension (A1) was determined spectrophotometrically at 640 nm. Three milliliter aliquots of the bacterial suspension were placed in contact with 1mL of the hydrocarbon phase (xylene). The mixture was vortexed for 2 min and allowed to stand. The aqueous phase (A2) was separated and the OD was redetermined at 640 nm. The hydrophobicity Index (HPBI) was calculated as (Nostro and Canatelli, 2004) defined below and categorized as per supplementary table 1.

3. Coaggregation of free probiotic suspension and *Candida albicans:* 100 mg of the lyophillised probiotic powder was weighed and dispersed in 10 mL normal saline solution (0.9% w/v) and kept for hydration for 5 minutes. After that the suspension was vortexed for 2 minutes to achieve uniform distribution. 1 mL aliquot (10^8cfu mL^{-1}) was withdrawn from the above probiotic suspension and mixed with 1 mL (10^7 cfu mL^{-1}) of *Candida albicans* suspension. The mixture was vortexed for at least 10 seconds and then a droplet of the suspension was placed on a glass slide and gram stained for visual observation of aggregates.

4. Ex vivo vaginal studies: Sterilisation of Vaginal samples - Porcine vaginal sections were placed in sufficient quantities of povidine-iodine solution (CIPLA) in a 15 mL culture tube

and inverted several times. The mucosa was transferred to sterile tubes containing phosphate buffer saline, (PBS, pH 6.8), which were inverted again several times to rinse off the povidine- iodine solution. The mucosa was then rinsed several times as explained above, with 70% ethanol. Final rinsing was done with PBS (pH 6.8) to remove ethanol and any other reagents remaining in the vaginal mucosa sample (Richard and Kaur 2005). The entire procedure was done in a laminar flow hood under aseptic conditions and all the apparatus and solutions used were sterile.

Table S1. Criteria for categorizing the hydrophobicity of bacteria.

S. No.	HPBI (%)	Category
1	Greater than 70%	Highly hydrophobic
2	50-70%	Moderately hydrophobic
3	Less than 50%	Low hydrophobicity

Table S2. Composition of simulated vaginal fluid.

Composition	Weight (g/L)
Sodium chloride	3.51
Potassium chloride	1.40
Calcium hydroxide	0.222
Bovine serum albumin	0.018
Lactic acid	2.00
Acetic acid	1.00
Glycerol	0.16
Urea	0.40
Glucose	5.00

Table S3. Grading of inflammatory reactions of vagina.

**Erythema Formation**	
No erythema	0
Very slight erythema (barely perceptible)	1
Well defined erythema	2
Moderate to severe erythema	3

Severe erythema (beef redness) to eschar formation preventing grading of erythema	4
Maximum possible score:4	

Results:

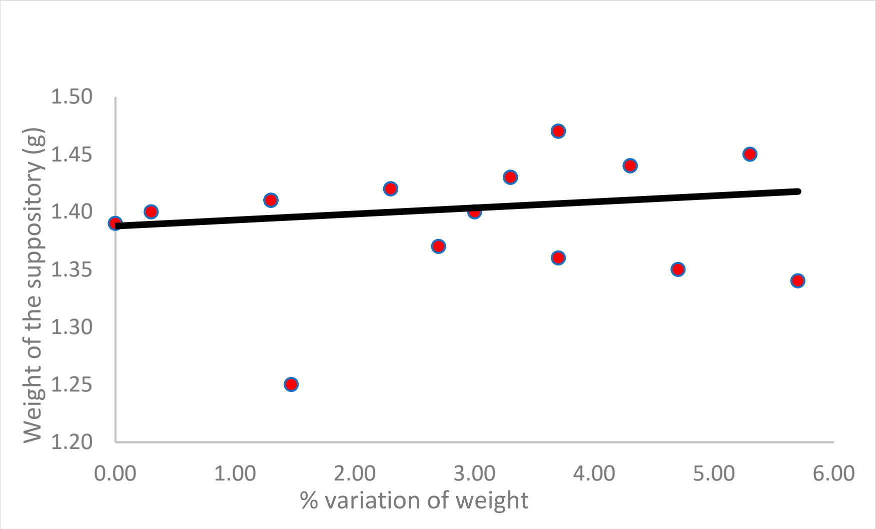

Figure S1. Weight of the emulgel suppository versus % difference from average.

a

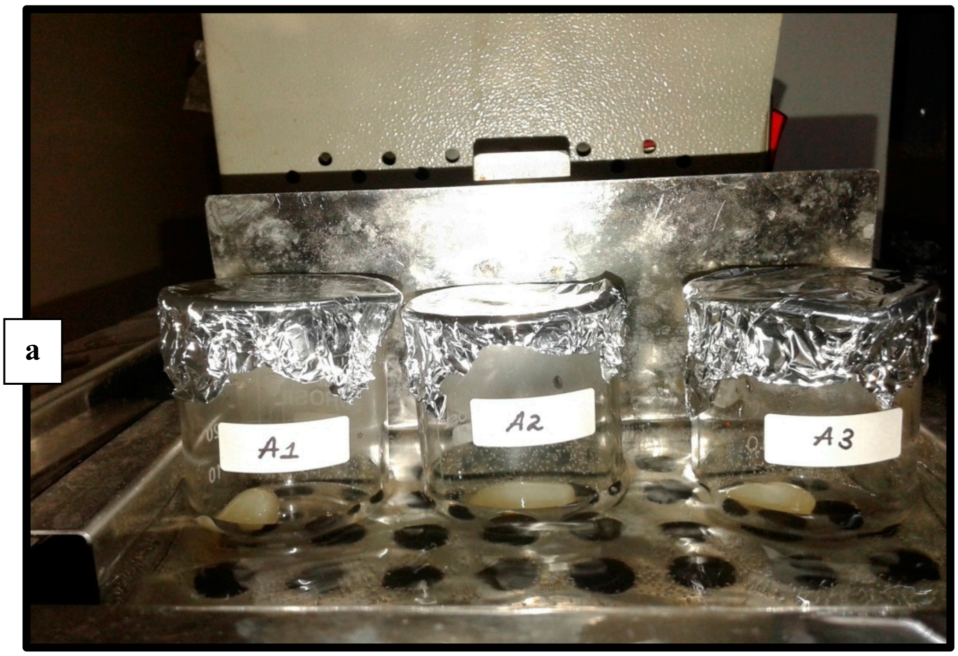

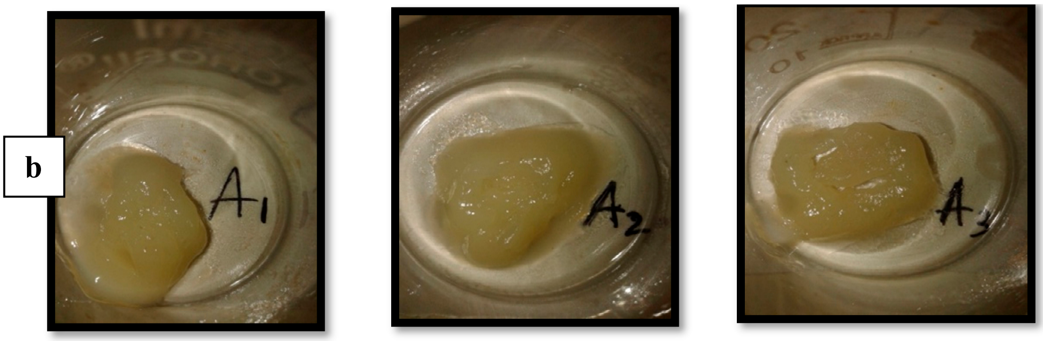

Figure S2. Melting of emulgel suppositories placed at 37 ^{0}C (a) Onset at 10 minutes (b) Complete melting at 2h.

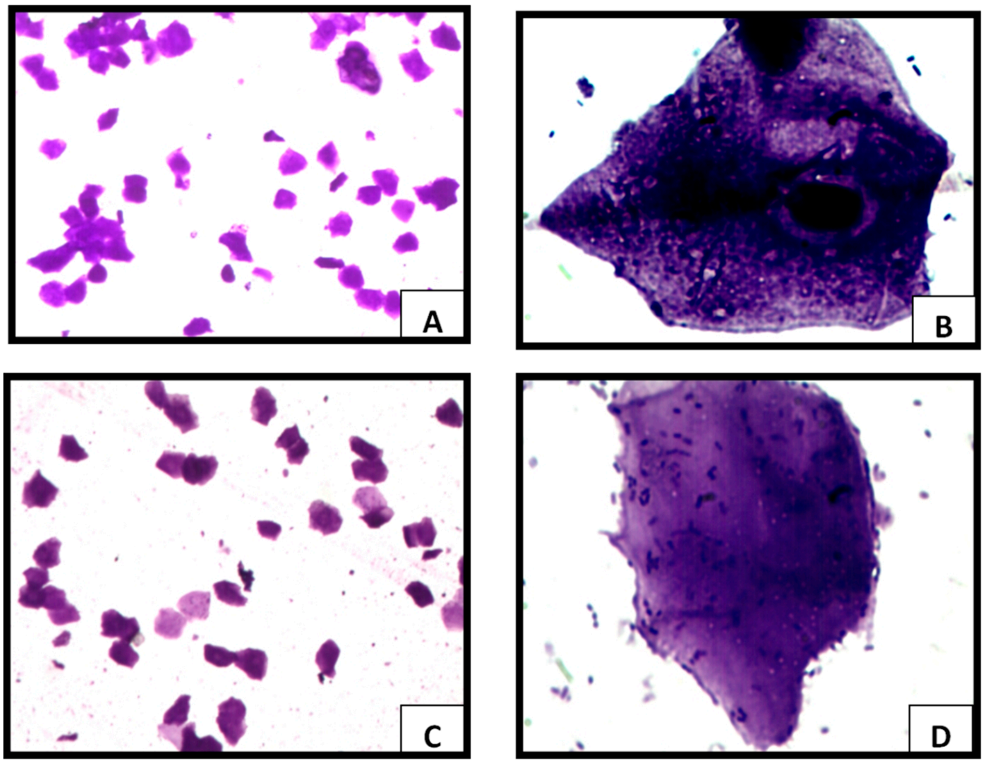

Figure S3. Vaginal smear showing nucleated epithelial cells in rats prior to estrogen treatment (A) 10×, (B) 100×, and after 6 days of estrogen treatment: (C) 10×, (D) 100×.
